# Supplementary material for: Patterns of Intron Gain and Loss in Fungi
Source: PLoS Biol. 2004 Nov 30;2(12):e422. doi: 10.1371/journal.pbio.0020422 (PMC532390; doi:10.1371/journal.pbio.0020422)
Supplement: Table S1 — Also available at http://genes.mit.edu/NielsenEtAl/. (4.3 MB ZIP). [file pbio.0020422.st001.zip › NielsenEtAl/html/793.html]

AN1413.1.NCU02082.1.MG00739.1.FG04907.1


```
 CLUSTAL W (1.82) Multiple Sequence Alignments - Introns Inserted


Sequence 1: NCU02082.1	1487 aa
Sequence 2: FG04907.1	1436 aa
Sequence 3: MG00739.1	1475 aa
Sequence 4: AN1413.1	1339 aa
Alignment Length: 1552 aa
Number Identitical Residues: 411 aa
Alignment Score (without introns) 26182


MG00739.1 	MM0APRVHAS2KTSTGWVNASANPVMQCYTELTPPSAVTHSLTLPFTSPKSNNLVVAKSS
NCU02082.1	--~-------~--------------MQCYTELTPPTAVTHSVTLQLVPGQGTNLAVAKAS
FG04907.1 	--~-------~--------------MQAYTELAAPSAVTHSLTASLTSATANNLVVAKGS
AN1413.1  	--~-------~--------------MQCYTELISPTGVTHALAVPFLSATANNLIVARTS
          	                         **.**** .*:.***:::  : .  ..** **: *

MG00739.1 	LLQIFATRLVPAELD------GTSQSAKATHN--YDTK-LNDDEGLEASFLGGDAAIIRS
NCU02082.1	LLQIFKTKVVSAEIDTYATLNGTNTSSKAAAAGRYDSRLVNDDDGFEASFLGGDNIAARA
FG04907.1 	LLQIFTTKAISAEFD---PENQQALPTKPEPE--FDHR-ANDDDGLESSFLGGETMIVKT
AN1413.1  	LLQIFSLRDVS----------LSALDTEVRPA---------------------------Q
          	*****  : :.               ::                                

MG00739.1 	DR-NHTKLVLVAEFPLSGTITGLARVKANATKTSNGNGAGSSSSGGDFLLIAFKDAKLSL
NCU02082.1	DRANSAKLVLVAEVTLPGTMTGLARIKK---------PSGSSSGGADCLLLSFRDARLSL
FG04907.1 	DRTNNTKLVLVAELPLSGAVTGLAKVK-----------TKHSKCGGEALLIAYKAAKLCM
AN1413.1  	HRQETCKLVLEREYQLPGTVTDICRVK-----------ILKTKSGGDAVLVAFRDAKLSL
          	.* :  ****  *  *.*::*.:.::*              :. *.: :*:::: *:*.:

MG00739.1 	VEWDPDRRSLETISIHYYEQNELQSSPWAAPLSDYVNFLVADPGSRCAALKFGARSLAII
NCU02082.1	VEWNVERNTLETVSIHYYEKEELVGSPWVAPLHQYPTLLVADPASRCAALKFSERNLAIL
FG04907.1 	AVWDPEKSTLETISIHYYEKEELHGAPWEVSFDEYANYLEADPGSRCAAFQFGSRNIAIL
AN1413.1  	VEWDPERYGLSTISIHYYERDDMTRSPWASDLSTCGSILSADPGSRCAIFQFGARSLAII
          	. *: ::  *.*:******::::  :**   :    . * ***.**** ::*. *.:**:

MG00739.1 	PFKQADGDIGMDDWDEELDGPRPAQEKPATAATNGTTDNVVEDTPYTPSFVLRLPNLDPA
NCU02082.1	PFKQPDEDMDMDNWDEELDGPRPKK-DLSGAVANGAST--IEDTPYSPSFVLRLSKLEAS
FG04907.1 	PFRQAEEDLEMDDWDEDLDGPRPVK-ETA-AVANGDSD--TVEPPYTPSFVLRLPLLDPS
AN1413.1  	PFHQPGDDLVMDDFGSEPDYENRVE-GNSRSHEAKDKDAAEYQTPYASSFVLPLTALDPS
          	**:*.  *: **::..: *  .  :   : :     .     :.**:.**** *. *:.:

MG00739.1 	LLHPVHLAFLYEYREPTFGILSSNITPSTYLAR--KDHLTYTVFTLDLQQKASTTILSVG
NCU02082.1	LLHPVHLAFLHEYRDPTIGVLSSTKTASNSLGH--KDHFTYMVFTLDLQQRASTTILAVN
FG04907.1 	LLHPVHFAFLHEYREPTFGILSSSQERAHSLGQ--KDHLTYKVFTLDLQQRASTTILSVT
AN1413.1  	VIHPISLAFLYEYREPTFGILYSQVATSHALLHERKDVVFYTVITLDLEQRASTTLLSVT
          	::**: :***:***:**:*:* *    :  * :. ** . * *:****:*:****:*:* 

MG00739.1 	GLPKDLTRVIALPAPVGGALLVGSNELIHIDQSGKANGVAVNPMTKSCTSFSLADQSDLG
NCU02082.1	GLPQDLFRVVALPAPVGGALLVGANELIHIDQSGKSNGIAVNPLTKQTTSFSLVDQADLD
FG04907.1 	DLPRDLFKILALPAPVGGALLIGENELIHVDQSGKANGVAVNSMARQITSFSLTDQADLN
AN1413.1  	RLPSDLFKVVALPPPVGGSLLIGSNELVHIDQAGKTNAVGVNEFSRQASSFSMTDQSDLA
          	 ** ** :::***.****:**:* ***:*:**:**:*.:.** :::. :***:.**:** 

MG00739.1 	LRLEGCMINVLSAEDGQFIIVLNDGRLATLVFHIDGRTVSGLKIKMVAPEAGGQLLQTSV
NCU02082.1	LRLEGCAIDVLAAELGEFLLILNDGRLGLITFRIDGRTVSGLSIKMIAPEAGGSVIQSRV
FG04907.1 	LRLEHCVVEQLHIENGELLLVLNDGQIGIVSFLIDGRTVSGLSIKMVTDENGGNVLKSRA
AN1413.1  	LRLENCVVERFSDDNGDLLLALSTGVFALVSFKLDGRSVSGISVRPLSGPSK-EFLASTA
          	**** * :: :  : *:::: *. * :. : * :***:***:.:: ::     ..: : .

MG00739.1 	SCLTRLGRNALFAGSDRGDSVVFGWNRKHNQVSKRKPKIQDPDLDLDIDYDDLEDDEDDD
NCU02082.1	TSLSRMGRSTMFVGSEEGDSVLLGWTRRQGQTQKRKSRLQDADLDLDLDDEDLEDDDD--
FG04907.1 	STASKLGKNTFFVGSEMGDSVVLGWTRKMGQEKRRKPRLIDTDIALDVDELDLEDDDDED
AN1413.1  	SSSAFLGNGKVFFGSESADSVLLGWSSASSATKKSFSGSTS---------NDESEDDAYE
          	:  : :*.. .* **: .***::**.   .  .:  .   .          * .:*:  .

MG00739.1 	DDLYADTEKTKA--TTSASTGETKTDDLIFRVHDLMVSIAPIRDVTFGKPPPPTDAERNT
NCU02082.1	DDLYGEESASPE--QAMSAAKAIKSGDLNFRIHDRLLSIAPIQKMTYGQPVTLPDSEEER
FG04907.1 	DDLYGTESAAAK--PAQALNGSGRSGELSFRIHDTLLSIAPIKDLTPGKTSFLPDSEEMT
AN1413.1  	DDLYSSAPAAMTDNPQNQPSNSSVAAFGDLRIHDRLSSPGPIRDIVLGRSS--EASSRDT
          	****.    :  ..          :    :*:** : * .**:.:. *:.     :..  

MG00739.1 	KDPAAVQSELQLVAVVGRDKASSLAIINREMTPVSIGRFEFPEARGLWTLSTQKPLPKPL
NCU02082.1	NS-EGVRSDLQLVCAVGRGKASALAIMNLAIQPKIIGRFEFPEARGFWTVCAKKPIPKTL
FG04907.1 	LS-DGVVSDLHLACIVGRGKAGSLAILNRNIQPKIIGRFEFPEARGFWTMSVKKPLPKAL
AN1413.1  	KD-----GVLELVAAQGSDEGGTMVIMKREVDPYLVASMAADTANSLWTVSLLP------
          	 .     . *.*..  * .:..::.*::  : *  :. :    *..:**:.         

MG00739.1 	QASNKNPKTAAATESILSAQYDQYMIVAKEDDDGFETSDVYALTAAGFETLSGTEFEPAA
NCU02082.1	VGDKGP----MNNDYDTSGQYHKFMIVAKVDLDGYETSDVYALTAAGFESLTGTEFEPAA
FG04907.1 	GGSAG-----VGDEYEAFGQHDKYMIVAKVDLDGYETSDVYALTGAGFETLKETEFDPAA
AN1413.1  	---------------DNNDQKRDYVILSKQEKPDKEESEVFVLEDK-LRPITAPEFNPNH
          	                   *  .::*::* :  . * *:*:.*    :..:. .**:*  

MG00739.1 	GFTIEAGTMGDHTKIIQVLKSEVRCYDGD1LGLTQIIPM~LDEETGHEPRATSASIADPY
NCU02082.1	GFTVEAGTMGKDSRILQVLKSEVRCYDGD1LGLSQIVPM~LDEETGAEPRVRTASIADPF
FG04907.1 	GFTVEAGTMGKQMRIIQVLKSEVRSYDGD1LGLTQILPM~LDEETGAEPRVTSASIVDPY
AN1413.1  	ELTVEIGTLASKSRVIQVLRNEVRSYD--~-------AV1WDEDDSDERVAVNATLVDPY
          	 :*:* **:... :::***:.***.**          .:  **: . *  . .*::.**:

MG00739.1 	LLIIRDDSSAFIAHVN-EDSEIEEIEKEDKIISSTKWSTGCLYADSKGAFAATQQTAKSP
NCU02082.1	LLLIRDDFSVFIAEMSPKLLELEEVEKEDQILTSTKWLAGCLYTDTSGVFADETVGKGT-
FG04907.1 	LLLIRDDSSLLLAQID-SNNELEEVEKMDATLQNTKWHAGCLYADTEGAFQFNANDKGE-
AN1413.1  	LAIIRDDSTLLLLQAD-DSGDLDEVTLSEDVVS-QKWLSACFYSDNAGFFTAPFA-----
          	* :**** : :: . . .  :::*:   :  :   ** :.*:*:*. * *          

MG00739.1 	KSTPTIMMFLLSAAGALY0IYALPDISRPVYVAEGLCYVPPYLSADYSARKGMARETISE
NCU02082.1	--KDNILMFLLSTSGVLY0IYRLPDLTKPVYVAEGLSYIPPGLSADYAARKGTAKESVAE
FG04907.1 	--TEKIMMFLLSSTGALH0VYALPDLSKPVYVAEGLSYVPPHLSADYTLRRGLAKETLRE
AN1413.1  	-----SILFLLNQDHQLY0VYRLPDFAV-ISVIEGVGCLPPILSTE-PPKRSTTRENVLQ
          	      ::***.    *: :* ***::  : * **:  :** **:: . ::. ::*.: :

MG00739.1 	ILVTDLGDTVFKSPHVI~LRHSNHDLTIYEPYRIAE--DSQSLTKILRLRKLPNPAVAKA
NCU02082.1	ILVADLGDTTHKSPYLI0LRHANDDLTLYQPYRLKATAGQP-FSKSLFFQKVPNSTFAKA
FG04907.1 	ILVADLGDTISQSPYLI~LRNQTDDLTIYEPIHHVRPGGESNLSAALSFKKMSNVTLATT
AN1413.1  	IAVVELGDSYSSLPFLI0LRTENDDLVVYKPFFTNS-----KELTGLRFLKEANHTLPKT
          	* *.:***:  . *.:* **  ..**.:*:*          .    * : * .* :...:

MG00739.1 	PEATNSEDPP--LMSRNMPLRACANIAGYSAVFMPGHSPSFLIKSAKATPKVIGLRGSGV
NCU02082.1	PEEKPADDDEPHNAQRFLPMRRCSNISGYSTVFLPGSSPSFILKTAKSSPRVLSLQGSGV
FG04907.1 	PAQTEDDDVE---QPRFMPMRRCANINGYSTVFLPGSSPSFVLKSSKSIPRVIGLQGLGI
AN1413.1  	PNTTDELQSE------MKPLRILPNIAGCSSIFMPGPSAGFIFRASTTSPHFIRLRGGFI
          	*  .   :          *:*  .** * *::*:** *..*:::::.: *:.: *:*  :

MG00739.1 	RAMSSFHTEGCERGFIYADSA~GVARVAQIPKDTSFSELGLSVKKVPLGIDADGIAYHSP
NCU02082.1	QAMSSFHTEGCEHGFIYADTN~GIARVTQIPTDSSYAELGLSVKKIPIGVDTQSVAYHPP
FG04907.1 	RGMSSFHTEGCDRGFIYADDK~GIARVTQFPSDTNFTELGISVKKVPLGSDVRGIAYHQP
AN1413.1  	KGLGCFDSP--DKGFAYLDSH0GLH-LAKLPEGTQLGYP-WIMRTVPIGQQIDKLTYVSA
          	:.:..*.:   ::** * *   *:  ::::* .:.       ::.:*:* :   ::*  .

MG00739.1 	TGVYVLTCSYWEPFELPKDDDYHCEWAKEN~ISFKPMVERSVLKVINPINWSDIWT~EEF
NCU02082.1	TQAYVVGCNDVEPFELPKDDDYHKEWAREN~ITFKPMVDRGVLKLLSGITWTVIDT~VEM
FG04907.1 	TGAYIAGCMTSEPFELPKDDDYHKEWAKET~LSFPPTMPRGVLKLISPITWTVIHD~IEL
AN1413.1  	SDTYVLGTCQRCEFRLPEDDELHPEWRNEE1ISFLPEVNQSSLKVVSPKTWSVIDS2YPL
          	: .*:        *.**:**: * ** .*  ::* * : :. **::.  .*: *     :

MG00739.1 	EQHEVAMCIRSLNLEVSQSTNERRQLITVGTAMCKGEDLPVRGGIYVFDLASVVPQKGRP
NCU02082.1	EPCETVLCVETLNLEVSESTNERKQLIAVGTALIKGEDLPTRGRVYVFDIADVIPEPGKP
FG04907.1 	ESCESIECMKTLHLEVSEDTKERRFLVAVGTAVSKGEDLPIRGRVHVYDIVTVIPEPGKP
AN1413.1  	EPAEHIMVMKTMSLEVSENTHERRDMIVVGTSLARGEDIPSRGCIYVFEVIEVVPDPEQP
          	*  *    :.:: ****:.*:**: ::.***:: :***:* ** ::*:::  *:*:  :*

MG00739.1 	ETDKKLKQVAKEEIPRGAVTSLSEIGTQGLMMVAQGQKTLVRGLQEDGKLPPVAFMDMNC
NCU02082.1	ETSKKLKLVAKEDIPRGAVTALSEVGTQGLMLVAQGQKCMVRGLKEDGTLLPVAFMDMNC
FG04907.1 	ETNRRLKAIAREDIPRGGVTAISEIGTQGLMLVAQGQKCMVRGLKEDGSLLPVAFLDMSC
AN1413.1  	ETNRRLKLIGKEPV-KGAVTALSEIGGQGFLIAAQGQKSMVRGLKEDGSLLPVAFMDMQC
          	**.::** :.:* : :*.**::**:* **:::.***** :****:***.* ****:**.*

MG00739.1 	YVTCVKELAGTGLCVMADAFKGVWFCGYT~EGPYKMMLFGKSSTNLECMNVDLLPDGKDL
NCU02082.1	YVTSVKELPGTGLCLMADAFKGVWFTGYT~EEPYKMMLFGKSSTRMEVLNADFLPDGKEL
FG04907.1 	HVSSARELSRTGLCLMADAFKGVWFAGYT~EEPYTFKVLGKSHGRLPVVVADFLPDGDDL
AN1413.1  	FVSVIKELKGTGMCIFGDAVKGLWFAGYS0EEPYKMSLFAKDLDYLEVLAADFLPDGNKL
          	.*:  :**  **:*::.**.**:** **: * **.: ::.*.   :  : .*:****..*

MG00739.1 	LIVAADSDGNLHVLQFDPEH1PKSLQGHLLLNRTTFSTGAHHPQKSLLLPTTDPRPS-TN
NCU02082.1	YIVASDADGHIHILQFDPEH1PKSLQGHLLLHRTTFNTGAHHPTSSLLLPAVYPNPSSLS
FG04907.1 	AIVAADVDGDLHILEFNPEH1PKSLQGHLLLHRTSFSVSPNPPSTTLLLPRTTPP----S
AN1413.1  	FIVVADSDCNLYVLQYDPED1PNSSNGDKLLNRSKFHTG-NFASTVTLLPRTLVSSERAM
          	 **.:* * .:::*:::**. *:* :*. **:*:.* .. : . .  *** .   ..   

MG00739.1 	QPSS------DAERQHILMASPTGVLAAVQPLSQSTYTRLSALASNLMASVPHHAALNPK
NCU02082.1	SNSE------ENSPHILLLASPTGVLATLRPLQENAYRRLSSLAVQLTNGLPHPAGLNPK
FG04907.1 	HPTP------QDPPHVLLLASSSGHLSSLIPLPETAYRRLLSVTNQLLPALTPHGGLNAK
AN1413.1  	SGSDKMDIDNTAPLHQVLVTSHNGSIGLVTCVPEESYRRLSALQSQLTNTLEHPCGLNPR
          	  : . . ..    : :*::* .* :. :  : : :* ** ::  :*   :    .**.:

MG00739.1 	AYRLPP--TSTRNQVAAVDISVGRAVVDGSLLARWAELASGRRAEVAGRAGFSG------
NCU02082.1	GYRLPSPSASASMQLPGVDAGIGRNIVDGKILERFLELGTGKRQEMAGRAGYVVGTGAHG
FG04907.1 	AHRLP-----VGTRTVGVEAAGGRAIVDGAVLARWAELSAAKRAEIAGKGGYDG------
AN1413.1  	AYRAVES-----------DASAGRGMLDSNLLLQYLDMSKQRKAEIAGRVGATE------
          	.:*   .           : . ** ::*. :* :: ::.  :: *:**: *         

MG00739.1 	----------------------------------AAEVRSELEAVLGWSI~MSYF-----
NCU02082.1	VNPAGSGKMMGGGGGLSLGGLRLNGLHGQEGGMEWEEVRGELGVVLGWSG~LGYF-----
FG04907.1 	----------------------------------VEELREELEGVLGWSE1LCYNKTSSS
AN1413.1  	-----------------------------------WEIRADLEAISGG-G~LGYL-----
          	                                    *:* :*  : *    : *      

MG00739.1 	----
NCU02082.1	----
FG04907.1 	LATH
AN1413.1  	----
          	
```
